# Prospects for Epigenetic Targeted Therapies of Bone and Soft-Tissue Sarcomas

**DOI:** 10.1155/2021/5575444

**Published:** 2021-07-26

**Authors:** Jun Wang, Arielle Elkrief, Wei Guo, Neerav Shukla, Mrinal Gounder, Marc Ladanyi

**Affiliations:** ^1^Musculoskeletal Tumor Center, Peking University People's Hospital, Beijing, China; ^2^Department of Pathology, Memorial Sloan Kettering Cancer Center, New York, NY, USA; ^3^Department of Pediatrics, Memorial Sloan Kettering Cancer Center, New York, NY, USA; ^4^Department of Medicine, Memorial Sloan Kettering Cancer Center, New York, NY, USA

## Abstract

Targeted therapies have revolutionized cancer treatment. It is well established that alterations of chromatin configuration and modifications affect tumorigenesis of some, possibly most, bone and soft-tissue sarcomas. As epigenetic regulators play a major role in the development of bone and soft-tissue sarcomas, epigenetic drugs provide a novel potential avenue for rational targeted therapies for these aggressive cancers. The present review summarizes the application of epigenetic drugs for clinical utilization in bone and soft-tissue sarcomas and provides an overview of clinical trials currently evaluating epigenetic therapies in this space.

## 1. Introduction

Historically, bone and soft-tissue sarcomas have been treated with combinations of surgery, chemotherapy, and radiation. Despite optimal locoregional therapy, 40% of patients with soft-tissue sarcoma develop metastases, and outcomes for patients with metastatic disease remain poor [1, 2]. Thus, new therapeutic strategies are clearly needed for this patient population.

Several lines of preclinical data suggest that epigenetic changes including DNA methylation and histone acetylation contribute to pathogenesis through modification of gene transcription [3]. Indeed, alterations of chromatin configuration and associated epigenetic modifiers have been implicated in the tumorigenesis of bone and soft-tissue sarcomas, and these results open the possibility of novel drugs targeting epigenetic modifications in this patient population [4–8]. The present review aims to provide an overview of the preclinical development of epigenetic-related targeted drugs and their respective clinical applications in bone and soft-tissue sarcomas. We analyze therapeutic targets involving each layer of epigenetic control, including at the level of DNA, histone, enzymatic complexes, and chromatin. We also highlight clinical trials investigating the use of epigenetic therapeutics in bone and soft-tissue sarcomas (Table 1).

## 2. DNA Methyltransferase Inhibitors

DNA methyltransferases (DNMTs) are a family of enzymes responsible for DNA methylation through the addition of a methyl group to the carbon atom number five (C5) of cytosine. Given the role of DNMTs in cellular differentiation, modifications in DMNT function have also been associated with tumorigenesis [9–12]. Five DNMT isoforms have been identified, including DNMT1, DNMT2, DNMT3a, DNMT3b, and DNMT3L. Among these isoforms, only DNMT1, DNMT3a, and DNMT3b have DNA methylation ability. While DNMT1 provides maintenance methyltransferase activity after DNA replication, DNMT3a and DNMT3b are *de novo* methyltransferases, which initiate DNA methylation marks on unmethylated DNA [9–11]. DNA methylation has been explored as a driver of tumorigenesis in Ewing sarcoma and central chondrosarcoma [13, 14].

Over the past decade, the cytidine analogues 5-Azacytidine/VIDAZA (AZA) and 5-Aza-2′-deoxycytidine/DACOGEN (DAC or decitabine) have been shown to induce hypomethylation in cancer cells, but have mostly been used in the context of hematologic malignancies. The next-generation DNA methyltransferase inhibitor (DNMTi) SGI-110 (guadecitabine) is a new small-molecule DNMTi agent whose resistance to cytidine deaminase has resulted in a longer half-life in an aqueous solution compared to first-generation DNMTis. The gradual cleavage of guadecitabine into decitabine leads to more prolonged and stable release of the drug, as opposed to decitabine's short-term peak in plasma concentration. Both AZA and decitabine have a short half-life, whereas guadecitabine which couples decitabine and deoxyguanosine, has a longer-acting epigenetic demethylation effect. Due to its superior pharmacokinetic characteristics, the novel DNMTi agent guadecitabine has been hypothesized to demonstrate superior antitumor activity. To date, guadecitabine has demonstrated encouraging clinical activity in phase I/II trials in patients with acute myeloid leukemia [15]. Modest clinical activity of guadecitabine has been reported in a phase II trial of patients with previously treated high-risk myelodysplastic syndrome, chronic myelomonocytic leukemia, and low-blast-count acute myeloid leukemia (NCT02197676) [16].

Building on the rationale for the use of guadecitabine in liquid malignancies, Fischer et al. compared the effect of AZA, decitabine, and guadecitabine on the inhibition of tumor proliferation in leiomyosarcoma cell lines. The authors found that guadecitabine was the most effective at decreasing cell survival in this preclinical model [4]. In an alveolar rhabdomyosarcoma (RMS) cell line, Darvishi et al. found that treatment with guadecitabine reduced cell proliferation as well as expression of FGFR4, a key receptor tyrosine kinase in RMS [5].

Beyond its ability to affect DNA methylation, guadecitabine also has also been shown to induce the expression of proinflammatory antigens on tumor cells, providing a rationale for its role in combination with immunotherapy agents [17–19]. Indeed, Coral et al. analyzed several tumor cell lines including the osteosarcoma cell line, MG63, and their results demonstrated the ability of guadecitabine to induce the expression of different immune-related antigens on human cancer cells. This effect could improve the immunogenicity of these tumors, with potential applications in combinations in chemoimmunotherapy for sarcoma based on these preclinical findings in this osteosarcoma model [19]. In the clinical setting, guadecitabine is currently under evaluation in patients with solid tumors with SDH loss including soft-tissue sarcomas (NCT03165721).

Combination treatment regimens including a DNMT inhibitor are another potential novel therapeutic strategy under evaluation. A multicenter phase II trial investigating the combination of AZA with the histone deacetylase inhibitor (HDACi) entinostat was performed in women with advanced hormone-resistant or triple-negative breast cancer (TNBC). Unfortunately, clinical benefit was minimal with only one partial response among 27 participants. In a subgroup analysis, patients who survived >20 months had a greater degree of global demethylation compared to those who had worse outcomes. This finding points to the possibility of demethylation status as a biomarker of response to these agents [20]. Several clinical trials showed limited therapeutic efficacy of this approach in breast, colon, and hematologic malignancies [20–22]. Further mechanistic studies and preclinical investigations in the sarcoma setting are needed in order to evaluate the combination of these two epigenetic targeting drug classes in bone and soft-tissue sarcomas.

## 3. EZH2 Inhibitors

Enhancer of Zeste Homolog 2 (EZH2) is a histone methyltransferase and the core enzymatic subunit of the polycomb repressive complex 2 (PRC2). EZH2 catalyzes the trimethylation of histone H3 at Lys 27 (H3K27me3), which can negatively regulate gene expression. It can also modulate cell cycle regulation, DNA damage repair, cell differentiation, and apoptosis. Aberrant expression of EZH2 has been detected in different sarcoma subtypes and has also been associated with poor prognosis [23–25]. Changchien et al. reported that poorly differentiated synovial sarcoma had higher expression of EZH2 compared to monophasic and biphasic synovial sarcomas and that EZH2 levels were found to correlate with the expression of Ki-67 and, as expected, H3K27me3 [25]. Furthermore, Yalcinkaya et al. validated these finding and showed that mutation or overexpression of EZH2 in soft-tissue sarcomas was associated with larger tumor size and worse clinical outcomes [26].

A potential clinical application of these findings is in the setting of epithelioid sarcoma, a rare histologic subtype of soft-tissue sarcoma occurring most often in adolescents and young adults with high propensity for local recurrence and distant metastases. For localized disease, surgical resection including amputations can be curative with a disease-free survival and overall survival rate of 54.5 months and 71%, respectively. However, local recurrence and distant metastases remain high [27]. In the advanced setting, outcomes are poor with median progression-free survival ranging from 3 to 6 months [28, 29]. INI1 (SMARCB1) is a tumor suppressor gene located on chromosome 22q11 that encodes a subunit of the SWI/SNF chromatin remodelling complex [30]. Loss of INI1 is common in epithelioid sarcoma and plays a crucial role in its sarcomatogenesis by impairing SWI/SNF function which causes aberrant PRC2 activity and tumor dependency on activating enhancer of zeste homolog 2 (EZH2) activity [30]. EZH2 inhibitors (EZH2i) have been shown to reduce tumor growth and promote apoptosis and autophagy in preclinical models across different tumor histologic types characterized by the presence of EZH2-activating mutations or altered EZH2 expression. Early activity was confirmed in clinical trials of tazemetostat in patients with solid tumors harboring dysregulation of EZH2 [31, 32]. A recent phase II trial demonstrated clinical benefit (tumor response or stable disease) in 26% of patients, with a median overall survival of 19 months [33]. This has led to FDA approval of tazemetostat for patients with metastatic or locally advanced epithelioid sarcoma not eligible for complete resection [34]. Tazemetostat has also demonstrated activity in poorly differentiated chordomas, an exceptionally rare subset of INI1-negative tumors. In an ongoing phase 1 study in pediatric patients, sustained responses have been observed in 2 out of 4 patients with chordoma [35]. NCT04705818 (not yet open) will evaluate the addition of durvalumab to tazemetostat in a cohort of patients with solid tumors, including patients with anthracycline-resistant soft-tissue sarcoma.

Interestingly, some patients with aberrant EZH2 or INI1 appear to be resistant to EZH2i therapy. In one study, autophagy was proposed as a possible mechanism of drug resistance to EZH2 inhibitor therapy [36]. Another potential mechanism of resistance of EZH2 inhibition therapy could be epigenetic crosstalk between methylation and acetylation. Huang et al. reported that gain of H3K27 acetylation (possibly MLL1-mediated) and oncogenic reprogramming influenced EZH2i response and limited the antitumor activity of EZH2 inhibitors [37]. Increased H3K27ac expression was also seen after EZH2i treatment and could be responsible for treatment resistance in this setting [31].

## 4. LSD1 Inhibitors

KDM1A (LSD1/BHC110) is the first flavin adenine dinucleotide- (FAD-) dependent lysine-specific demethylase identified to catalyze the demethylation of mono- and dimethylated K4 or K9 on histone H3 (H3K4me1/2 and H3K9me1/2) [38]. Aberrant overexpression of LSD1 has been observed in various human cancers and sarcomas and is closely associated with differentiation, proliferation, migration, invasion, and poor prognosis [39]. Bennani-Baiti et al. investigated the role of LSD1 expression in over 500 sarcoma tumor samples. Using gene expression profiling and immunohistochemistry, the authors demonstrated that LSD1 may be overexpressed in several histologic subtypes of bone sarcoma and STS, including chondrosarcoma, Ewing sarcoma, osteosarcoma, rhabdomyosarcoma, and synovial sarcoma, and this overexpression of LSD1 primarily showed nuclear localization [40]. Inhibiting LSD1 expression using small molecules results in suppression of tumor cell differentiation, proliferation, invasion, and migration [38–40]. Ewing sarcoma is characterized by EWSR1-FLI1 fusions, which act as an aberrant transcription factor. This fusion is crucial to the epigenetic pathogenesis of Ewing sarcoma through its participation in abnormal chromatin remodelling, via DNA binding domains of EWSR1-FL1 which recognize microsatellites with GGAA repeats, which, in turn, leads to recruitment of histone acetyltransferases to the nucleosome, causing loosening of the tertiary structure of DNA, enabling gene transcription [41]. LSD1 associates with EWSR1-FLI1 to alter gene expression and contribute to disease progression [41]. To date, multiple LSD1 inhibitors have been developed for the treatment of hematologic malignancies and solid tumors, including RG6016, GSK-2879552, INCB059872, IMG-7289, CC-90011, SP-2509, and SP-2577 [42, 43], and a clinical trial investigating SP-2577 in relapsed Ewing sarcoma (NCT03600649) is currently recruiting patients. In the preclinical setting, inhibition of KDM1A/LSD1 using SP-2509, a reversible inhibitor, suppressed cell growth in a Ewing sarcoma cell line [44, 45]. However, a second group of researchers found that inhibition of KDM1A catalytic demethylase activity was insufficient as a therapeutic strategy for Ewing sarcoma in 3D spheroid tissue cultures [46]. When analyzing mechanisms of resistance to LSD1 inhibitors in Ewing's sarcoma, Pishas and Lessnick reported that cells resistant to SP-2509 overexpressed the multidrug resistance genes ABCB1, ABCC3, and ABBC5 after SP-2509 treatment [47]. Even several months after SP-2509 withdrawal, SP-2509 resistance was still apparent, pointing to the irreversible nature of the drug-induced epigenetic states. Pishas and Lessnick concluded that SP-2509 resistance in Ewing sarcoma was not fully reversible or driven by direct mutation in KDM1A and, therefore, may have been due to other epigenetic mechanisms [47]. Combination regimens of LSD1 inhibitors and other epigenetic drugs may have more activity than LSD1 inhibitors alone. Haydn et al. found that the concomitant inhibition of LSD1 and HDAC synergistically induced cell death in RMS cells by shifting the ratio of pro- and antiapoptotic BCL2 family proteins in favor of apoptosis, engaging the intrinsic apoptotic pathway. These findings provide a rationale for evaluating a combination of LSD1 inhibitors with HDAC inhibitors in soft-tissue sarcoma [48].

## 5. Acetylation Modification

Acetylation of histones is an important modification in the control of gene expression [37]. Tumor suppressor genes are silenced mainly by increased HDAC activity and/or decreased activity of histone acetyltransferases (HATs) [49]. Upregulation of HDAC activity and downregulation of HAT activity can also control apoptosis-related genes [50]. Several HDAC inhibitors (HDACi) exist, either acting as pan-HDAC inhibitors or as compounds specific for individual class 1 and 2 HDAC enzymes [51]. Many clinical trials of epigenetic acetylation-related drugs have been launched for hematologic diseases and solid tumors. Thus, a large number of HDAC inhibitors have been developed and extensively investigated for their antitumor potential. Notably, the FDA has approved Zolinza (vorinostat), Istodax (romidepsin), and Beleodaq (belinostat) to treat cutaneous and peripheral T-cell lymphoma and Farydak (panobinostat) for multiple myeloma. Although the development of HDACi for treatment of bone and soft-tissue sarcomas lags behind its application in hematologic malignancies, there are several clinical trials evaluating the use of HDACi in treatment of soft-tissue sarcoma (i.e., NCT02282917, NCT01175109, NCT03345485, NCT00918489, NCT01294670, NCT04308330, NCT01879085, NCT01132911, and NCT00937495).

### 5.1. Vorinostat and EDO-S101

Vorinostat is one of the most widely used HDAC inhibitors. HDAC inhibitors may cause DNA damage and then abrogate DNA double-strand break repair. Specifically, vorinostat can relax the chromatin structure by increasing the acetylation of histones, giving DNA-damaging agents easier access to chromatin. A multicenter phase II trial for refractory soft-tissue sarcomas recruited 40 patients with refractory STS receiving vorinostat after failure of 1 (*n* = 8, 20%), 2 (*n* = 10, 25%), or ≥3 (*n* = 22, 55%) previous lines of chemotherapy [52]. In this study, median progression-free survival and overall survival were 3.2 and 12.3 months, respectively. PFS rates at 3 and 6 months were 58% and 29%, respectively, and a subgroup of six patients had long-lasting stable disease for up to ten cycles. Although objective response to vorinostat was low for the heavily pretreated population, a small subgroup of patients had prolonged stable disease [52]. A phase II trial combining vorinostat and the proteasome inhibitor bortezomib did not demonstrate significant activity in patients with STS [53]. However, since some patients benefit from treatment with vorinostat, combination regimens continue to be explored [54, 55]. Several clinical trials seeking to evaluate the therapeutic effect of vorinostat combination regimens are underway (NCT00918489, NCT01294670, NCT04308330, NCT01879085, NCT01132911, and NCT00937495).

EDO-S101 (tinostamustine) is a novel first-in-class fusion molecule composed of the alkylator bendamustine and the HDAC-inhibitor vorinostat [56]. So far, EDO-S101 has been predominantly investigated in hematological malignancies, and preclinical and early clinical results of its use in multiple myeloma are encouraging [57, 58]. Investigations of EDO-S101 in solid malignant tumors are ongoing, such as a phase I/II clinical trial of EDO-S101 in patients with solid cancers, including soft-tissue sarcomas, small-cell lung cancer, triple-negative breast cancer, ovarian cancer, and endometrial cancer (NCT03345485).

### 5.2. AR-42 (OSU-HDAC42)

AR-42 (OSU-HDAC42) is a novel phenylbutyrate-based class I/IIB HDAC inhibitor which is mainly used in the treatment of hematologic malignancies [59, 60]. Preclinical studies demonstrated that AR-42 may be active in musculoskeletal sarcomas. Murahari et al. analyzed the therapeutic effect of AR-42 on osteosarcoma *in vitro* and revealed that it was a potent inhibitor of osteosarcoma cell viability [60]. Furthermore, this agent was also found to induce cell apoptosis via the activation of the intrinsic mitochondrial pathway through activation of caspase 3/7 and suppression of AKT signaling and of BCL-XL. Murahari et al. compared two HDAC inhibitors, vorinostat (suberoylanilide hydroxamic acid (SAHA)) and AR-42, on the osteosarcoma cells and found AR-42 to be more potent [60]. AR-42 has so far not been evaluated clinically in the treatment of bone and soft-tissue sarcomas although a proof-of-concept study (NCT02282917), recruiting patients with vestibular schwannoma and meningiomas, has been launched.

### 5.3. Panobinostat (LBH589)

Panobinostat (LBH589) is a novel cinnamic hydroxamic acid histone deacetylase inhibitor, active on HDAC class I and II isoforms at nanomolar concentrations [61]. Disease control with panobinostat was first observed in a patient with Ewing sarcoma [62]. The French Sarcoma Group launched a phase II trial of panobinostat in 47 patients with advanced pretreated STS of various subtypes. In this trial, nine patients (20%) were progression free at 3 months and six were progression free at 6 months. Unfortunately, no patient had a partial response and only 17 patients (36%) showed stable disease status as their best response [63]. These results demonstrate that although some patients can show disease stabilization, therapeutic efficacy of single-agent panobinostat in advanced STS is limited.

In the combination setting, Bauer et al. explored a regimen of panobinostat with imatinib in 11 patients with refractory metastatic gastrointestinal stromal tumors in a phase I study. One of 11 evaluable patients had a partial response (PR) and 7 had stable disease (SD), while 3 had progressive disease (PD) [64]. Thomas et al. investigated a panobinostat and epirubicin combination regimen in a phase I trial including 20 patients with sarcomas. 12/20 patients benefited (1 PR, 11 SD, median OS 8.3 months), including 8/14 who had previously progressed on anthracycline therapy. This trial demonstrated that the treatment regimen of panobinostat combined with epirubicin was well tolerated and can show activity in anthracycline-resistant tumors (NCT00878904) [65].

Despite a strong rationale for the use of epigenetic modifying drugs in bone and soft-tissue sarcomas, the therapeutic potential of these agents has yet to be fully harnessed. Moreover, mechanisms of resistance to these drugs remain to be elucidated, and optimal combination strategies need to be defined. Exploration of combination regimens are of particular interest, as are correlative biomarker studies to better define subgroups of patients who stand to benefit from epigenetic therapeutic approaches. In summary, epigenetic therapies are emerging as an active and promising area of precision oncology in bone and soft-tissue sarcomas.

## Figures and Tables

**Table 1:** Selected clinical trials of epigenetic drugs for bone and soft-tissue sarcomas.

Clinical trial identifier	Drug	Mechanism	Status	Patient population	Phase
NCT02601937	Tazemetostat (EPZ-6438)	Selective small molecule inhibitor of the histone-lysine methyltransferase EZH2	Recruiting	(i) Rhabdoid tumors; atypical teratoid rhabdoid tumor (ATRT); malignant rhabdoid tumor (MRT); rhabdoid tumor of kidney (RTK); selected tumors with rhabdoid features; INI1-negative tumors; epithelioid sarcoma; epithelioid malignant peripheral nerve sheath tumor; extraskeletal myxoid chondrosarcoma; myoepithelial carcinoma; renal medullary carcinoma; other INI1-negative malignant tumors (e.g., dedifferentiated chordoma); synovial sarcoma	I
NCT03165721	Guadecitabine (SGI-110)	DNA methyltransferase inhibitor (DNMT1 inhibitor)	Completed	(i) Wild-type gastrointestinal stromal tumors; paraganglioma gastrointestinal stromal tumors; renal cell renal neoplasms; pheochromocytoma	II
NCT03600649	SP-2577	LSD1 inhibitor	Recruiting	(i) Relapsed or refractory Ewing sarcoma	I
NCT03514407	INCB059872	LSD1 inhibitor	Completed	(i) Relapsed Ewing sarcoma	I
NCT02282917	AR-42(OSU-HDAC42)	Histone deacetylase inhibitor	Active, not recruiting	(i) Vestibular schwannoma; meningioma; acoustic neuroma; neurofibromatosis type 2	1
NCT01175109	Imatinib + LBH589	LBH589, a histone deacetylase inhibitor	Completed	(i) Newly diagnosed and recurrent chordoma	I
NCT03345485	Tinostamustine (EDO-S101)	First-in-class alkylating histone deacetylase inhibition (HDACi) fusion molecule	Active, not recruiting	(i) Small-cell lung cancer; soft-tissue sarcoma; triple-negative breast cancer; ovarian cancer; endometrial cancer	I/II
NCT00918489	Vorinostat	Histone deacetylase inhibitor	Completed	(i) Metastatic soft-tissue sarcoma	II
NCT01294670	Vorinostat and etoposide	Histone deacetylase inhibitor	Completed	(i) solid tumors; relapsed/refractory sarcomas	I/II
NCT04308330	Vorinostat in combination with vincristine, irinotecan, and temozolomide	Histone deacetylase inhibitor	Recruiting	(i) Ewing sarcoma; rhabdomyosarcoma; Wilms tumor; neuroblastoma; hepatoblastoma; germ-cell tumor	I
NCT01879085	Vorinostat in combination with gemcitabine and docetaxel	Histone deacetylase inhibitor	Recruiting	(i) Metastatic or locally advanced soft-tissue sarcoma	I/II
NCT01132911	Vorinostat and bortezomib	Histone deacetylase inhibitor	Completed	(i) Lymphoma; sarcoma; Wilms tumor; neuroblastoma	I
NCT00937495	Vorinostat and bortezomib	Histone deacetylase inhibitor	Completed	(i) Recurrent or advanced adult soft-tissue sarcoma	II
